# Dyadic patterns of subjective cognitive decline and longitudinal cognitive outcomes

**DOI:** 10.1002/alz.087053

**Published:** 2025-01-03

**Authors:** Michelle L. Houston, Panpan Zhang, Dandan Liu, Kimberly R. Pechman, Timothy J. Hohman, Angela L. Jefferson, Katherine A. Gifford

**Affiliations:** ^1^ Vanderbilt Memory & Alzheimer’s Center, Vanderbilt University Medical Center, Nashville, TN USA; ^2^ Department of Biostatistics, Vanderbilt University Medical Center, Nashville, TN USA; ^3^ Vanderbilt Memory and Alzheimer’s Center, Vanderbilt University Medical Center, Nashville, TN USA; ^4^ Department of Neurology, Vanderbilt University Medical Center, Nashville, TN USA

## Abstract

**Background:**

Subjective cognitive decline (SCD) may provide an early indicator of Alzheimer’s disease. Dyadic SCD, the self/patient’s in relation to an informant/loved one’s endorsement of SCD, may better reflect underlying Alzheimer’s pathology. We related dyadic SCD discrepancies to longitudinal changes of cognitive aging.

**Methods:**

Vanderbilt Memory and Aging Project participants (cognitively unimpaired 71%, n = 537, 69.3±10.1 years, mean follow‐up time 5.9±2.8 years) and their informants completed the Everyday Cognition (ECog) questionnaire and Cognitive Complaint Questionnaire (CCQ). Dyadic discrepancy (self‐reported score minus informant‐reported score) was calculated and split into 3 levels (i.e., self>informant, informant>self, and dyadic concordance). Participants completed a diagnostic interview, neuropsychological testing, and multimodal brain MRI at each timepoint. Linear mixed‐effects regression models related baseline dyadic category to diagnostic conversion and longitudinal neuropsychological and neuroimaging outcomes. Models were adjusted for age, sex, self‐reported race/ethnicity, baseline diagnosis, *APOE‐*ε4 status, depression symptoms (Geriatric Depression Scale), and follow‐up time. A false discovery rate (FDR) correction for multiple comparisons was applied.

**Results:**

Dyadic discrepancy on the ECog did not relate to diagnostic conversion. Compared to dyadic concordance, individuals with greater self‐SCD on the CCQ related to diagnostic conversion (β = 2.96, p = 0.04). In secondary models stratified by diagnosis, cognitively unimpaired participants with greater self‐SCD were more likely to diagnostically convert (β = 6.48, p = 0.02). Compared to dyadic concordance, individuals with greater informant‐SCD on the ECog were associated with worsening executive functioning (β = ‐0.06, pFDR = 0.02), Boston Naming Test (BNT) (β = ‐0.42, pFDR<.001), and animal naming (β = ‐0.44, pFDR = 0.01). Compared to dyadic concordance, individuals with greater informant‐SCD on the CCQ were associated with worsening executive functioning (β = ‐0.08, pFDR = 0.01), memory (β = ‐0.07, pFDR = 0.01), and BNT (β = ‐0.53, pFDR<.001), with stronger associations in the mild cognitive impairment subgroup. Neither ECog nor CCQ dyadic discrepancy related to neuroimaging markers (pFDR>0.10).

**Conclusions:**

The direction of dyadic discrepancy related differently to diagnostic conversion and cognitive performance. Greater self‐reported SCD related to greater likelihood of diagnostic conversion in cognitively unimpaired participants. However, greater informant‐reported SCD related to worsening cognitive performance. Results highlight that dyadic patterns of SCD differentially predict prognosis and incorporation of an informant provides important information. Further analyses will examine dyadic discrepancy and longitudinal biomarker patterns.

